# Effects of risk-based multifactorial fall prevention on health-related quality of life among the community-dwelling aged: a randomized controlled trial

**DOI:** 10.1186/1477-7525-5-20

**Published:** 2007-04-26

**Authors:** Sari Vaapio, Marika Salminen, Tero Vahlberg, Noora Sjösten, Raimo Isoaho, Pertti Aarnio, Sirkka-Liisa Kivelä

**Affiliations:** 1 Institute of Clinical Medicine, Family Medicine, University of Turku, Turku, Finland; 2 Satakunta Central Hospital, Pori, Finland; 3 Unit of Family Medicine, Turku University Hospital, Turku, Finland; 4 Institute of Clinical Medicine, Biostatistics, University of Turku, Turku, Finland; 5 Pori Health Center, Pori, Finland; 6 Nordic School of Public Health, Gothenburg, Sweden

## Abstract

**Background:**

This study aimed to assess the effects of a risk-based, multifactorial fall prevention programme on health-related quality of life among the community-dwelling aged who had fallen at least once during the previous 12 months.

**Methods:**

The study is a part of a single-centre, risk-based, multifactorial randomised controlled trial. The intervention lasted for 12 months and consisted of a geriatric assessment, guidance and treatment, individual instruction in fall prevention, group exercise, lectures on themes related to falling, psychosocial group activities and home exercise. Of the total study population (n = 591, 97% of eligible subjects), 513(251 in the intervention group and 262 in the control group) participated in this study. The effect of the intervention on quality of life was measured using the 15D health-related quality of life instrument consisting of 15 dimensions. The data were analysed using the chi-square test or Fisher's exact test, the Mann-Whitney U-test and logistic regression.

**Results:**

In men, the results showed significant differences in the changes between the intervention and control groups in depression (p = 0.017) and distress (p = 0.029) and marginally significant differences in usual activities (p = 0.058) and sexual activity (p = 0.051). In women, significant differences in the changes between the groups were found in usual activities (p = 0.005) and discomfort/symptoms (p = 0.047). For the subjects aged 65 to 74 years, significant differences in the changes between the groups were seen in distress (p = 0.037) among men and in usual activities (p = 0.011) among women. All improvements were in favour of the intervention group.

**Conclusion:**

Fall prevention produced positive effects on some dimensions of health-related quality of life in the community-dwelling aged. Men benefited more than women.

## Background

The assessment of health-related quality of life (HRQOL) has become common in clinical trials, although in fall prevention trials it is still less commonly used. In fall prevention studies HRQOL has usually been classified as a secondary outcome and only parts (for example physical or functional ability) of the quality of life (QOL) instruments have been used [1-3]. Prevention of falls in later life is a key public health priority Presenting and measuring outcomes of quality of life is relevant because physical frailty and fall-related injuries are two of the biggest threats to QOL or HRQOL, or to functioning of older people in general. Due to consequences of falls such as fractures and fear of falling the physical, psychological and social functional abilities decrease which can have a considerable impact on perceived QOL [4].

Randomized controlled trials (RCTs) involving fall prevention interventions including the assessment of quality of life among the community-dwelling aged [5-14] have shown some positive effects on quality of life only in two RCTs [5,6]. These RCTs have comprised exercise and Comprehensive Geriatric Assessment (CGA) programmes and have shown significant improvements in physical function, physical health, mental health, social function, vitality and energy/fatigue of HRQOL in the intervention groups. Interventions comprising exercise and information programmes, performed among nursing home residents and hospitalised participants, have shown improvements in role-physical, role-emotional, pain and general health of HRQOL [7,8].

A risk-based, multifactorial randomised controlled trial among the previously fallen community-dwelling aged was conducted in Pori, in western Finland. The purpose of the trial was to assess the effects of a multifactorial fall prevention programme on the incidence of falls and injurious falls, selected risk factors of falling, physical, psychological and social functional abilities, HRQOL, use of health services and mortality [15]. A fall was defined as an unexpected event where a person falls to the ground from an upper level or same level [16]. In this study HRQOL was conceptually defined as part of the overall QOL by WHO (the World Health Organisation): "An individual's perception of their position in life in the context of the culture and value systems in which they live and in relation to their goals, expectations, standards and concerns. A broad ranging concept affected in a complex way by the person's physical health, psychological state, personal beliefs, social relationships and their relationship to salient features of their environment" [17-19]. This study is part of the randomised controlled trial and the paper reports the effects of fall prevention programme on the dimensions of HRQOL measured with the 15D instrument in a risk group of the community-dwelling aged.

## Methods

### Study population

**Figure 1:**
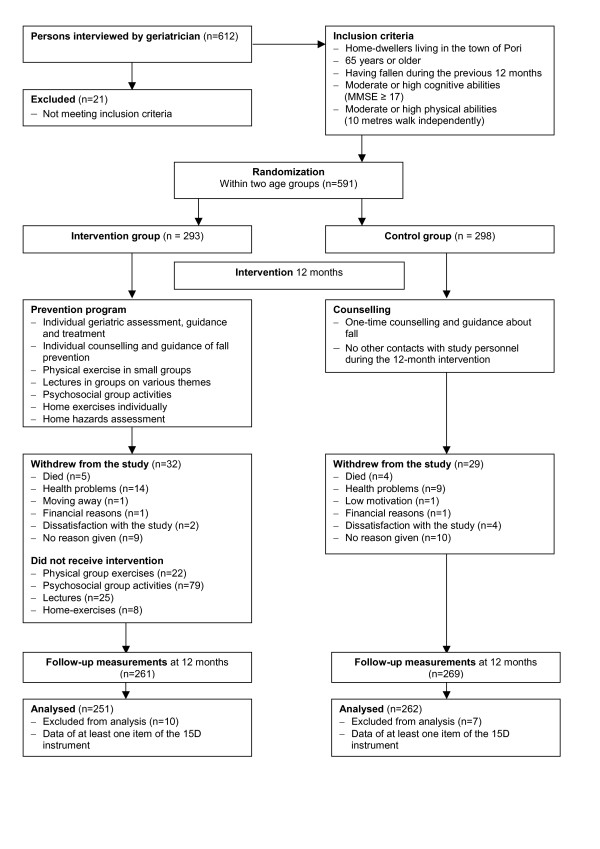
Progression of the study.

The participants were 65 years or older, who had fallen at least once during the previous 12 months, had moderate or good physical abilities (able to walk 10 meters independently with or without walking aids) and cognitive abilities (Mini-Mental State Examination MMSE ≥17) and were living at home or in sheltered housing (sheltered home or homelike living). The sample size, based on a power calculation, was estimated based on the results of previous fall prevention studies showing that every third fall or fall injury could be prevented [20-22]. The sample was estimated to consist of at least 458 persons, representing about 10% of the persons aged over 65 years or older who had fallen during the previous year in the town of Pori in western Finland, where this trial was conducted (total population of over 65-year-old persons was 13547 at the baseline of the intervention). The persons willing to participate and fulfilling the inclusion criteria (n = 591) were randomised into an intervention group (IG) (n = 293) and a control group (CG) (n = 298) separately in two age groups (65–74 years and ≥75 years) after the baseline assessment using consecutively numbered sealed envelopes (Figure 1). The design and the predictors of adherence to this randomised controlled trial have been described in more detail elsewhere [15,23].

### HRQOL measurement

HRQOL measurements in both groups were completed immediately before and after the 12-month intervention. The 15D health-related quality of life instrument was used as a secondary outcome measure in the trial. QOL deserves more attention in fall prevention RCTs and that is why this paper focuses only the effects of HRQOL. The 15D is a generic, comprehensive, multi-dimensional (physical, mental and social well-being), standardised and self-administered instrument and it is described in a greater detail in Sintonen 1994, 2001. It can be used to elicit both a profile and single index scores and it includes the following 15 dimensions: mobility, seeing (vision), hearing, breathing, sleeping, eating, speech (communication), elimination, usual activities, mental function, discomfort/symptoms (pain), depression, distress, vitality and sexual activity. The 15D items have five response categories [24,25].

### Intervention for fall prevention

The one-year intervention consisted of seven parts and was based on an individual risk factor analysis. The intensity of the activities was planned separately for each participant and increased progressively according to his/her health status. 1. A thorough individual geriatric assessment, guidance and treatment (recommendations concerning risk medications and visual, hearing and nutritional problems) were done by an experienced geriatrician. 2. Participants received individual guidance on fall prevention (e.g. importance of peroral calcium, hip protectors, home safety) by a trained public health nurse. 3. Physical group exercise was performed twice a month supervised by a physiotherapist. For the exercise groups the subjects were divided into three levels according to their physical functional abilities at baseline assessments (balance, muscle strength and respiratory function). Each exercise session began with warm up exercises (5–10 min), followed by exercises designed to improve lower leg muscle strength, balance and coordination (30 min) and ended up by cool-down exercises (5–10 min). Exercises could be performed in a sitting or standing position according to the subject's health and functional status. 4. Lectures on falling-related themes once a month were given by health professionals. The subjects in the intervention group were offered lectures on preventive aspects of falling, such as walking aids, nutrition in old age, home hazards and physical exercise and overall fall prevention. 5. Psychosocial group activities once a month were held by nursing students. Subjects were divided into two groups according to their psychological health, amount of depressive symptoms, feelings of loneliness and level of social activity. Those having few contacts with other people and feeling themselves lonely and whose sum score was over 10 in the Geriatric Depression Scale (GDS) (20) were advised to join a smaller "support" group. All the others were advised to join a bigger psychosocial group. Activities included discussions on different themes and actual events, group singing, quizzes, reading poems and a summer party. 6. The subjects were advised to perform physical exercises similar to those performed in groups at home three times a week. The subjects were given a brochure based on the exercise class content and encouraged to record the amount of their physical activity in the physical exercise diaries daily. 7. Home hazards assessment with a control visit approximately at six months was performed by trained nursing students. The assessment of the home environment consisted of questions about lighting, stairs, thresholds, corridors, floors, carpets, furniture and availability of handrails. Written suggestions for modifications were given to each subject and an additional home visit was performed to reinforce the modifications. The control group attended one counselling session at the baseline and had no other contacts with study personnel during the 12-month intervention [15].

### Ethics

The permission to conduct the study was issued by the chief physician of Pori Health Center and ethics approval was obtained from the Ethics Committee of Satakunta Hospital District. The participants gave written informed consent, and the study was conducted in accordance with the guidelines of the Declaration of Helsinki.

### Data analyses

The data were analysed on an intention-to-treat basis, to provide a realistic indication of the generalisability and effectiveness of the intervention [26]. Differences in the categorical sociodemographic variables between the IG and CG groups were analysed using the chi-square test or Fisher's exact test. The Mann-Whitney U-test was used to test the differences in the continuous variables between the groups. In the analysis of HRQOL outcome variables, the baseline differences between the groups were assessed using logistic regression. During the follow-up, the changes in HRQOL items were examined by logistic regression analyses with generalised estimation equations (GEE) to account for correlations between repeated measurements [27]. Cumulative logistic models were used for ordinal dependent items consisting of three categories and binary logistic models for dependent items having two categories. Differences in the changes between IG and CG were tested with interaction terms between the group and measurement. The results of logistic models were quantified by calculating cumulative odd ratios (COR) and odds ratios (OR) with their 95% confidence intervals (95% CI). Corresponding analyses were also performed in two age groups (65–74 years and ≥75 years). All analyses were carried out separately for men and women. P-values of less than 0.05 were considered statistically significant, and P-values between 0.05 and 0.080 marginally significant. SAS System for Windows, version 9.1 (SAS Institute Inc., Cary, NC) was used for statistical analyses.

The statistical methods used were chosen because they are appropriate methods to analyse the differences between the groups. Chi-square test or Fisher's exact test are basic statistical methods for analysing the differences between the categorical variables. Due to skewed distributions continuous, variables were analysed using non-parametric Mann-Whitney U-test. HRQOL outcome variables had two or three categories and the baseline differences between the groups were assessed using binary and cumulative logistic regression. For longitudinal data of HRQOL variables logistic regression analyses with generalized estimation equations to account for correlations between repeated measurements were used.

## Results

### Data collection

Participants with baseline and follow-up data of at least one item of the 15D were included in the analyses (n = 513). Of the 513 subjects, 251 (36 men and 215 women) belonged to IG and 262 (46 men and 216 women) belonged to CG. A total of 78 subjects dropped out (42 in IG and 36 in CG) during the 12 months follow-up.

The 15D was used to elicit profiles of participants. For statistical analyses the items were combined from 5 categories into 3 categories because of the small numbers of observations in the categories 4 and 5. The categories 3 to 5 were combined except for some items. The items of depression, distress and vitality were placed into 2 categories (the categories 2 to 5 were dichotomised) among men and the items of eating and speech among women. Among men, the items of eating and speech could not be used at all. It was not reasonable to recode the categories from 5 to 2 in all profiles without loosing some information. In the subgroup analyses among younger subjects, the item on depression was not included and the item on eating was divided into 2 categories among women.

### Dropout analyses

Among men, there was a difference between those who completed the study (n = 82) and those who dropped out (for reasons other than death) (n = 11) in MMSE (Median, lower quartile-upper quartile) (28.0, 26.0–29.0 and 26.0, 24.0–28.0, respectively) (p = 0.042). Cognitive functional ability was better among those completing the study compared to those who dropped out. Of those completing the study, 84% lived with a spouse or some other person and 16% lived alone, while the corresponding proportions among the dropouts were 45% and 55% (p = 0.008).

Among women, there were differences between those who completed the study (n = 431) and the dropouts (n = 58) in median age (72.0, 68.0–76.0 and 77.0, 73.0–84.0) (p < 0.001), MMSE (28.0, 26.0–29.0 and 27.0, 24.0–28.0) (p = 0.006), the Geriatric Depression Scale (GDS) scores (4.0, 1.0–8.0 and 5.0, 2.0–11.0) (p = 0.031) and the ability to manage activities of daily living (ADL) (32.0, 31.0–32.0 and 30.0, 25.0–32.0) (p < 0.001). Those who completed the study were younger and had better cognitive, mental and physical abilities compared to the dropouts.

### Baseline characteristics and participation rates

The intervention and control groups (n = 513) did not differ from each other in the baseline characteristics (Additional file 1). The majority of the participants (84%) were female, and 96% lived at home. The mean participation rate (the number of attended sessions divided by the number of sessions offered during the intervention period) of IG subjects were 65% among men and 63% among women in group exercises, 27% and 38% in lectures and 23% and 29% in psychosocial group activities, respectively. Men performed home exercises on an average of 2.5 (SD 2.2) and women 2.6 (2.0) times per week.

### Differences in changes in HRQOL

Differences in the changes between IG and CG were found in depression (p = 0.017) and distress (p = 0.029) and marginal differences in usual activities (p = 0.058) and sexual activity (p = 0.051) among men (additional file 2). In women, the analyses showed differences in the changes between the groups in usual activities (p = 0.005) and discomfort/symptoms (p = 0.047) (additional file 3.). These differences in changes were in favour of IG.

The analyses were also done by age groups 65–74 or ≥75 years. There were differences in changes among men in distress (p = 0.037) and among women in usual activities (p = 0.011) in the age group of 65–74 years. These differences in the changes were also in favour of IG.

## Discussion

This study is part of the randomised controlled trial, which is the largest risk-based, multifactorial fall prevention programme conducted in Finland. The aim of this paper was to assess the effects of fall prevention intervention on the dimensions of HRQOL measured with the 15D instrument in a risk group of the community-dwelling aged. The trial has several strengths. Randomisation was successful, and the intervention aimed to apply the best current evidence of fall prevention. The preventive methods were multifactorial, individually tailored and based on an individual risk factor assessment by a geriatrician, nurse and physiotherapist. The duration of the intervention was long and the effects of some outcomes will be followed up for five years. The outcome assessments were wide and the methods are suitable for primary care with some modification. The large sample size also enabled analysis of the effects in subgroups of participants. However, when implementing a trial in a small town, contamination of the control group by family members, friends or newspapers must be taken into account [15]. Attrition is a potential problem in studies where outcome measures are obtained over time. The sample size was estimated using power calculations. The dropout analyses showed that over a half of the men who did not complete the study lived alone and had a lower MMSE than study participants. The women who dropped out were older and had worse cognitive, psychological and functional abilities than the participants. Hence the subjects most likely to benefit from the intervention were not reached.

The health and HRQOL of the participants in this study were good at baseline (most answers of the participants were in categories signifying the best HRQOL). Thus, it is difficult to produce a change during the intervention. We combined the 15D items into 3 and even 2 categories because the frequencies in the categories four and five were too small for statistical analyses. It was noteworthy that men had more favourable changes in HRQOL than women, even though the male sample was considerable smaller than that of women. Men probably benefited from the physical exercise and group activities of the intervention. This may also have increased their social contacts. An increase in social contacts and changes in the social roles of men may at least partly explain the effects of the programme on depressive symptoms [28,29] and distress among men.

HRQOL instruments, different from that used in this study, namely SF-36 (The 36-Item Short-Form Health Survey), RAND-36 (36-Item Health Survey) and Euroqol (EQ-5D, European Quality of Life Instrument) were used in the previous studies, which were not multifactorial [5,14]. Thus, it is not easy to compare their results to ours. Structured quality of life questionnaires may also be geographically or culturally sensitive and inadequate in capturing all perspectives of the subjects' experiences [11]. In our study QOL is the secondary outcome measure of the trial and deserves more attention. Bacause of that the manuscript focuses on the effects of QOL. The 15D instrument was used to elicit profiles of participants and it is recommended by the Washington Panel and is valid, reliable, sensitive and responsive to change and generalisable at least in western-type societies. The 15D is particularly valid for deriving quality-adjusted life years (QALY's), which is applicable to cost effective analyses planned to be performed later. The characteristics and the validity of the instrument verified before the beginning of the study to make sure that results are reliable. Conceptually, the 15D subscribes to the definition of health by WHO, and it is available in several languages for population and clinical economic evaluation studies. It has been developed in Finland and validated among Finnish people but it has not been designed especially for the aged [24,25].

The 15D and most other HRQOL instruments used among older people have commonly measured health status, functional ability, impact of illness on people's lives or behaviour and influences on HRQOL rather than HRQOL itself [30-32]. Many valid instruments have been designed for measuring HRQOL in working-aged populations or among those suffering from a certain disease. They may not measure all dimensions of HRQOL, and it is important to assess the content of the instrument before application to older people [33-36]. However, it is likely that the participants' experiences may be best addressed by using both quantitative measures and qualitative methods, such as theme or in-depth interviews. In order to obtain results based on qualitative methods, random samples should be interviewed in clinical trials, even though additional economic resources are needed for that.

The analyses were performed for men and women separately because we were interested in knowing whether the effects of the intervention differ between men and women. The results were different and that is why they are also reported separately for men and women. The findings indicated improvement on the following dimensions of HRQOL: depression, distress, usual activities and sexual activity in men and usual activities and discomfort/symptoms in women in the intervention group compared with the control group. The subgroup analyses were planned before the beginning of the study. Two age groups (65–74 and ≥75 years) showed positive changes in distress among men aged 65–74 years and in usual activities among women aged 65–74 years in the IG. The results showed improvements in the intervention groups compared with the control groups, and they are supported by several previous intervention studies [5-8]. These interventions, single-based, exercise-oriented or Comprehensive Geriatric Assessment-oriented, had positive effects on the HRQOL of the community-dwelling aged in such domains as physical functioning, physical health, vitality, energy/fatigue, mental health and social functioning [5,6]. Some other fall prevention trials have not shown significant improvements in HRQOL [9,12-14].

The results of this study cannot be generalised to the unselected aged population. The intervention was targeted to persons with at last one previous fall, and the results represent the effects of a prevention program on a risk group. The social dimension of HRQOL is currently highlighted, particularly among the aged but it is usually lacking in assessments or has been measured too narrowly [37,38]. The 15D instrument contains four items about the psychological dimension and only one item about the social dimension. This lack has been compensated for in our trial by using additional questions about the psychosocial domains of HRQOL. The results on these domains will be analysed later.

Fall prevention may affect quality of life by different mechanisms. The group activities included in the programme may directly improve quality of life. Decrease in falls and fall injuries can also be a significant factor in improvement of quality of life. Furthermore maintenance of functional abilities and social activities by decreasing fall injuries may have a positive effect on quality of life. If the participants consider the prevention too demanding, requiring e.g. significant changes in health and other habits, the effects on the QOL may be negative. The effects of the intervention on falls and fall injuries have not yet been assessed. Thus, we do not know, if a reduced amount of injuries have been a factor affecting the positive results on HRQOL. We may say that our results on HRQOL show that the participants have not considered the intervention too demanding. The intervention was based mainly on group activities. We think that the more positive results among men compared to women may be due to group activities increasing social contacts of the participants. Older Finnish women have more social contacts and activities than older Finnish men [39]. Thus increasing social contacts may have more positive effects on men than on women.

## Conclusion

The measurement of quality of life is important because fall prevention intervention may have multi-level effects on HRQOL. The fall prevention intervention implemented here produced positive effects on some dimensions of HRQOL in a risk group of the community-dwelling aged. Men benefited more than women.

## Competing interests

The author(s) declare that they have no competing interests.

## Authors' contributions

S-LK planned and organised the multifactorial prevention trial. SV and MS conceptualised the design of this study together with S-LK. Statistical analyses and interpretation of data were made by SV, MS and TV. Writing and critical revision of the manuscript were accomplished by SV, MS and S-LK, and TV, NS, RI and PA participated in the writing and revising. Funding was obtained by S-LK together with RI and PA. Supervision and decision making to submit for publication were the tasks of SV, MS and NS. All authors read and approved the final manuscript.

## Supplementary Material

Additional file 1 Baseline characteristics in the intervention and control groups by gender. Click here for file

Additional file 2 Health related quality of life measured with 15D-instrument at baseline and after 12-month intervention in intervention and control groups among men. Click here for file

Additional file 3 Health related quality of life measured with 15D-instrument at baseline and after 12-month intervention in intervention and control groups among women. Click here for file
