# Supplementary material for: Effects of risk-based multifactorial fall prevention on health-related quality of life among the community-dwelling aged: a randomized controlled trial
Source: Health Qual Life Outcomes. 2007 Apr 26;5:20. doi: 10.1186/1477-7525-5-20 (PMC1868017; doi:10.1186/1477-7525-5-20)
Supplement: Additional file 1 — Baseline characteristics in the intervention and control groups by gender. [file 1477-7525-5-20-S1.doc]

**Table 1: Baseline characteristics in the intervention and control groups by gender.**

|  | **Intervention group**  n = 251 | | **Control group**  n = 262 | |
| --- | --- | --- | --- | --- |
|  | Men  n = 36 (14)  n (%) | Women  n = 215 (86)  n (%) | Men  n = 46 (18)  n (%) | Women  n = 216 (82)  n (%) |
| **Age*** , years | 72.0 (69.0-76.0) | 72.0 (68.0-76.0) | 73.5 (70.0-77.0) | 71.0 (68.0-76.0) |
| **Age**  65-74  ≥75 | 24 (67)  12 (33) | 140 (65)  75 (35) | 27 (59)  19 (41) | 148 (69)  68 (32) |
| **Marital status**  Single  Married or co-habiting  Widowed, divorced or judicial separation | 3 (8)  28 (78)  5 (14) | 13 (6)  81 (38)  121 (56) | 1 (2)  38 (83)  7 (15) | 16 (7)  94 (44)  106 (49) |
| **Education**  Less than basic  Basic  More than basic | 1 (3)  26 (72)  9 (25) | 2 (1)  153 (71)  60 (28) | 1 (2)  38 (83)  7 (15) | 2 (1)  151 (70)  63 (29) |
| **Living circumstances**  Living alone  Living with a spouse or another person | 5 (14)  31 (86) | 132 (61)  83 (39) | 8 (17)  38 (83) | 119 (55)  97 (45) |
| **Living place**  Home  Sheltered housing | 36 (100) | 207 (96)  8 (4) | 46(100) | 201 (93)  15 (7) |
| **MMSE*** | 28.0 (27.0-29.0) | 28.0(27.0-29.0) | 27.0(26.0-29.0) | 28.0 (26.0-29.0) |
| **GDS*** | 3.0 (2.0-7.0) | 4.0 (2.0-8.0) | 3.0 (1.0-8.0) | 4.0 (1.0-8.0) |
| **ADL*** | 31.5 (31.0-32.0) | 32.0 (30.0-32.0) | 32.0 (31-32) | 32.0 (31.0-32.0) |

*Median (lower quartile – upper quartile)

MMSE = Mini-Mental State Examination, GDS = Geriatric Depression Scale, ADL = Activities of daily living

No statistically significant differences between the groups were found either among men or women.
