# Supplementary material for: Effects of risk-based multifactorial fall prevention on health-related quality of life among the community-dwelling aged: a randomized controlled trial
Source: Health Qual Life Outcomes. 2007 Apr 26;5:20. doi: 10.1186/1477-7525-5-20 (PMC1868017; doi:10.1186/1477-7525-5-20)
Supplement: Additional file 2 — Health related quality of life measured with 15D-instrument at baseline and after 12-month intervention in intervention and control groups among men. [file 1477-7525-5-20-S2.doc]

**Table 2: Health related quality of life measured with 15D-instrument at baseline and after 12-month intervention in intervention and control groups among men**

| **Dimensions (13) of 15D-instrument and categories** | **Intervention group** | | | **Control group** | | | Interaction P-value* |
| --- | --- | --- | --- | --- | --- | --- | --- |
|  | Baseline  n (%) | Follow-up  n (%) | COR (95% CI) | Baseline  n (%) | Follow-up  n (%) | COR (95% CI) |  |
| **Mobility**  No difficulties  No difficulties indoors, slight difficulties outdoors  No help needed indoors, considerable difficulties outdoors or help needed - unable to move | 23 (66)  10 (29)  2 (6) | 26 (74)  7 (20)  2 (6) | 1.5 (0.8 - 2.8) | 30 (65)  11 (24)  5 (11) | 29 (63)  13 (28)  4 (9) | 1.0 (0.6 - 1.5) | 0.304 |
| **Seeing (vision)**  No difficulties  Slight difficulties  Considerable difficulties - almost or completely blind | 29 (81)  4 (11)  3 (8) | 26 (72)  7 (19)  3 (8) | 0.7 (0.3 - 1.4) | 31 (67)  10 (22)  5 (11) | 35 (76)  5 (11)  6 (13) | 1.4 (0.8 - 2.6) | 0.135 |
| **Hearing**  No difficulties  Slight difficulties  Considerable difficulties - completely deaf | 20 (57)  13 (37)  2 (6) | 22 (63)  10 (29)  3 (9) | 1.2 (0.7 – 2.0) | 23 (50)  20 (43)  3 (7) | 28 (61)  13 (28)  5 (11) | 1.4 (0.9 - 2.0) | 0.665 |
| **Breathing**  No difficulties  Shortness of breath during heavy work or sports  Shortness of breath when walking on flat ground - breathing difficulties in rest | 23 (68)  7 (21)  4 (12) | 25 (74)  6 (18)  3 (9) | 1.3 (0.8 - 2.2) | 25 (54)  13 (28)  8 (17) | 30 (65)  11 (24)  5 (11) | 1.6 (1.0 - 2.6) | 0.612 |
| **Sleeping**  No difficulties  Slight difficulties  Considerable difficulties - severe sleeplessness | 18 (55)  12 (36)  3 (9) | 24 (73)  6 (18)  3 (9) | 2.0 (1.0 - 4.3) | 21 (48)  15 (34)  8 (18) | 22 (50)  15 (34)  7 (16) | 1.1 (0.7 - 1.9) | 0.210 |
| **Elimination**  No difficulties  Slight difficulties  Considerable difficulties - no control | 17 (49)  16 (46)  2 (6) | 23 (66)  8 (23)  4 (11) | 1.7 (0.7 - 3.9) | 16 (36)  25 (57)  3 (7) | 20 (46)  21 (48)  3 (7) | 1.4 (0.7 - 2.6) | 0.631 |
| **Usual activities**  No difficulties  Slight difficulties  Considerable difficulties - unable to manage | 24 (69)  9 (26)  2 (6) | 28 (80)  4 (11)  3 (9) | 1.7 (0.9 - 3.3) | 35 (78)  9 (20)  1 (2) | 32 (71)  11 (24)  2 (4) | 0.7 (0.4 - 1.3) | 0.058 |
| **Mental function**  No difficulties  Slight difficulties  Considerable difficulties - permanently confused | 15 (43)  18 (51)  2 (6) | 13 (37)  21 (60)  1 (3) | 0.9 (0.5 - 1.6) | 22 (49)  21 (47)  2 (4) | 22 (49)  23 (51)  0 (0) | 1.1 (0.5 - 2.2) | 0.628 |
| **Discomfort/symptoms**  No discomfort or symptoms  Mild discomfort or symptoms  Marked - unbearable discomfort or symptoms | 8 (23)  25 (71)  2 (6) | 12 (34)  16 (46)  7 (20) | 1.0 (0.5 - 2.2) | 9 (20)  28 (62)  8 (18) | 13 (29)  25 (56)  7 (16) | 1.4 (0.8 - 2.6) | 0.457 |
| **Depression**  No depressive symptoms  Low, moderate - high amount of depressive symptoms | 27 (77)  8 (23) | 34 (97)  1 (3) | 10.1 (1.5 - 67,0)^†^ | 29 (64)  16 (36) | 33 (73)  12 (27) | 1.5 (0.9 - 2.7)^†^ | 0.017 |
| **Distress**  No feelings of distress  Slight feelings of distress  Moderate - high feelings of distress | 23 (66)  12 (34) | 32 (91)  3 (9) | 5.6 (1.6 - 19.3) ^†^ | 33 (73)  12 (27) | 35 (78)  10 (22) | 1.3 (0.7 - 2.5) ^†^ | 0.029 |
| **Vitality**  Healthy and energetic  Slightly or moderately weary, tired or feeble - totally exhausted | 19 (54)  16 (46) | 24 (69)  11 (31) | 1.8 (0.9 - 3.7) ^†^ | 18 (40)  27 (60) | 21 (47)  24 (53) | 1.3 (0.7 - 2.4) ^†^ | 0.469 |
| **Effect of state of health on sexual activity**  No adverse effect  Slight adverse effect  Considerable effect - sexual activity impossible | 8 (30)  8 (30)  11 (41) | 14 (52)  6 (22)  7 (26) | 2.3 (1.0 - 5.1) | 17 (47)  9 (25)  10 (28) | 17 (47)  6 (17)  13 (36) | 0.9 (0.5 - 1.4) | 0.051 |

Interaction P-value* between group and measurement; COR = Cumulative Odds Ratio for change within group during

the 12-month follow-up (higher functioning compared to lower functioning); OR^†^ = Odds Ratio
