# Supplementary material for: Effects of risk-based multifactorial fall prevention on health-related quality of life among the community-dwelling aged: a randomized controlled trial
Source: Health Qual Life Outcomes. 2007 Apr 26;5:20. doi: 10.1186/1477-7525-5-20 (PMC1868017; doi:10.1186/1477-7525-5-20)
Supplement: Additional file 3 — Health related quality of life measured with 15D-instrument at baseline and after 12-month intervention in intervention and control groups among women. [file 1477-7525-5-20-S3.doc]

**Table 3: Health related quality of life measured with 15D-instrument at baseline and after 12-month intervention in intervention and control groups among women**

| **Dimensions (15) of 15D-instrument and categories** | **Intervention group** | | | **Control group** | | | Interaction P-value* |
| --- | --- | --- | --- | --- | --- | --- | --- |
|  | Baseline  n (%) | Follow-up  n (%) | COR  (95% CI) | Baseline  n (%) | Follow-up  n (%) | COR  (95% CI) |  |
| **Mobility**  No difficulties  No difficulties indoors, slight difficulties outdoors  No help needed indoors, considerable difficulties outdoors - unable to move | 110 (51)  81 (38)  23 (11) | 132 (62)  53 (25)  29 (14) | 1.3 (1.1 - 1.7) | 115 (54)  81(38)  18 (8) | 126 (59)  69 (32)  19 (9) | 1.2 (0.9 - 1.5) | 0.397 |
| **Seeing (vision)**  No difficulties  Slight difficulties  Considerable difficulties - almost or completely blind | 156 (73)  41 (19)  18 (8) | 159 (74)  35 (16)  21 (10) | 1.0 (0.8 - 1.4) | 156 (73)  38 (18)  20 (9) | 160 (75)  38 (18)  16 (8) | 1.1 (0.8 - 1.5) | 0.768 |
| **Hearing**  No difficulties  Slight difficulties  Considerable difficulties - completely deaf | 133 (62)  66 (31)  16 (7) | 144 (67)  59 (27)  12 (6) | 1.3 (1.0 - 1.6) | 133 (63)  63 (30)  16 (8) | 140 (66)  62 (29)  10 (5) | 1.2 (0.9 - 1.5) | 0.731 |
| **Breathing**  No difficulties  Shortness of breath during heavy work or sports  Shortness of breath when walking on flat ground - breathing difficulties in rest | 104 (49)  78 (37)  30 (14) | 117 (55)  73 (34)  22 (10) | 1.3 (1.0 - 1.7) | 121 (57)  67 (31)  25 (12) | 137 (64)  55 (26)  21 (9.86) | 1.4 (1.0 - 1.7) | 0.820 |
| **Sleeping**  No difficulties  Slight difficulties  Considerable difficulties - severe sleeplessness | 62 (29)  93 (44)  58 (27) | 80 (38)  81 (38)  52 (24) | 1.3 (1.0 - 1.7) | 51 (24)  95 (45)  65 (31) | 66 (31)  78 (37)  67 (32) | 1.2 (0.9 - 1.5) | 0.442 |
| **Eating**  No difficulties  Slight difficulties  Need of help from other person | 211 (98)  4 (2) | 212 (99)  3 (1) | 1.3 (0.4 - 4.9) ^†^ | 209 (97)  6 (3) | 211 (98)  4 (2) | 1.5 (0.5 - 4.8) ^†^ | 0.890 |
| **Speech**  No difficulties  Slight difficulties  Considerable difficulties - unable to speak | 202 (94)  13 (6) | 207 (96)  8 (4) | 1.7 (0.8 - 3.6) ^†^ | 202 (94)  14 (7) | 207 (96)  9 (4) | 1.6 (0.9 - 2.9) ^†^ | 0.931 |
| **Elimination**  No difficulties  Slight difficulties  Considerable difficulties - no control | 95 (44)  87 (41)  33 (15) | 106 (49)  74 (34)  35 (16) | 1.2 (0.9 - 1.5) | 102 (48)  79 (37)  33 (15) | 105 (49)  54 (25)  55 (26) | 0.9 (0.7 - 1.1) | 0.133 |
| **Usual activities**  No difficulties  Slight difficulties  Considerable difficulties -unable to manage | 134 (63)  63 (29)  17 (8) | 150 (70)  47 (22)  17 (8) | 1.4 (1.0 - 1.8) | 153 (71)  50 (23)  13 (6) | 138 (64)  61 (28)  17 (8) | 0.7 (0.5 – 1.0) | 0.005 |
| **Mental function**  No difficulties  Slight difficulties  Considerable difficulties - permanently confused | 99 (46)  113 (53)  3 (1) | 91 (42)  116 (54)  8 (4) | 0.8 (0.6 - 1.1) | 97 (46)  110 (52)  6 (3) | 86 (40)  116 (55)  11 (5) | 0.8 (0.6 - 1.1) | 0.796 |
| **Discomfort/symptoms**  No discomfort or symptoms  Mild discomfort or symptoms  Marked - unbearable discomfort or symptoms | 24 (11)  125 (58)  65 (30) | 42 (20)  114 (53)  58 (27) | 1.4 (1.1 - 1.8) | 34 (16)  134 (64)  43 (20) | 42 (20)  114 (54)  55 (26) | 0.9 (0.7 - 1.29) | 0.047 |
| **Depression**  No depressive symptoms  Low, moderate - high amount of depressive symptoms | 129 (60)  74 (34)  12 (6) | 147 (68)  60 (28)  8 (4) | 1.5 (1.1 – 2.0) | 116 (55)  87 (41)  10 (5) | 141 (66)  64 (30)  8 (4) | 1.6 (1.2 - 2.1) | 0.644 |
| **Distress**  No feelings of distress  Slight, moderate - high feelings of distress | 132 (62)  77 (36)  5 (2) | 161 (75)  50 (23)  3 (1) | 1.9 (1.3 - 2.7) | 133 (62)  73 (34)  7 (3) | 150 (70)  57 (27)  6 (3) | 1.4 (1.0 - 1.9) | 0.248 |
| **Vitality**  Healthy and energetic  Slightly or moderately weary, tired or feeble - totally exhausted | 82 (38)  118 (55)  15 (7) | 115 (54)  88 (41)  12 (6) | 1.8 (1.4 - 2.3) | 90 (42)  107 (50)  16 (8) | 109 (51)  89 (42)  15 (7) | 1.4 (1.1 - 1.8) | 0.215 |
| **Effect of state of health on sexual activity**  No adverse effect  Slight adverse effect  Considerable effect - sexual activity impossible | 61 (78)  8 (10)  9 (12) | 64 (82)  7 (9)  7 (9) | 1.3 (0.7 - 2.4) | 65 (72)  13 (14)  12 (13) | 65 (72)  16 (18)  9 (10) | 1.1 (0.7 - 1.6) | 0.605 |

Interaction P-value* between group and measurement; At baseline significant difference were found between the groups in discomfort/symptoms (p<.012) and marginally significant difference in usual activities (p<.073); COR = Cumulative Odds Ratio for change within group during the 12-month follow-up (higher functioning compared to lower functioning); OR^†^ = Odds Ratio
